# Comparison of Physicochemical Properties of Starches from Flesh and Peel of Green Banana Fruit

**DOI:** 10.3390/molecules23092312

**Published:** 2018-09-11

**Authors:** Zheng Li, Ke Guo, Lingshang Lin, Wei He, Long Zhang, Cunxu Wei

**Affiliations:** 1 Key Laboratory of Crop Genetics and Physiology of Jiangsu Province/Key Laboratory of Plant Functional Genomics of the Ministry of Education, Yangzhou University, Yangzhou 225009, China; yzulizheng@163.com (Z.L.); 18115657147@163.com (K.G.); 18252713442@163.com (L.L.); hewei930312@163.com (W.H.); zhanglong@yzu.edu.cn (L.Z.); 2 Co-Innovation Center for Modern Production Technology of Grain Crops of Jiangsu Province/Joint International Research Laboratory of Agriculture & Agri-Product Safety of the Ministry of Education, Yangzhou University, Yangzhou 225009, China

**Keywords:** green banana, flesh, peel, starch, physicochemical properties

## Abstract

Green banana fruit is an important starch resource that consists of flesh and peel. The physicochemical properties of flesh starch have been widely studied; however, those of peel starch have hardly been studied, leading to the waste of peel. In this study, the physicochemical properties of the starches from the flesh and peel of green banana fruit were investigated and compared. The dry flesh and peel had 69.5% and 22.6% starch content, respectively. The starch had oval and irregular granules with eccentric hila. Their starches had similar bimodal size distribution; the volume-weighted mean diameter was approximate 17 μm, and the peel starch had a slightly smaller granule size than the flesh starch. The maximum absorption wavelength was higher in peel starch than in flesh starch. The apparent amylose content of flesh and peel starch was 21.3% and 25.7%, respectively. The flesh and peel starches both exhibited B-type crystalline structures and had similar relative crystallinity, short-range ordered degrees, and lamellar structures. The swelling power was similar between flesh and peel starches, but the water solubility was higher in peel starch than in flesh starch at 95 °C. The peel starch had a higher gelatinization temperature than flesh starch, but their gelatinization temperature range and enthalpy were similar. Both flesh and peel starches showed a diphasic hydrolysis dynamic, but peel starch had higher resistance to porcine pancreatic α-amylase hydrolysis than flesh starch. The contents of rapidly digestible starch, slowly digestible starch, and the resistant starch of flesh and peel were 1.7%, 4.3%, 94.1% and 1.4%, 3.4%, 95.2%, respectively, for native starch, and 73.0%, 5.1%, 21.9%, and 72.3%, 4.5%, 23.2%, respectively, for gelatinized starch.

## 1. Introduction

Starch is synthesized in amyloplasts or chloroplasts, consists of amylose and amylopectin, and exists as semi-crystalline granules in plants. Usually, starch is stored in cereal seeds, fruits, and some metamorphic roots and stems [1,2,3,4,5]. Starch not only plays an important role in plant growth and development, it is also widely used as food for people’s daily life and raw material for food and nonfood processing industries. Starches from different plant resources have different morphologies, granule sizes, structures, and physicochemical properties, which determine their different applications [1,2,3,4,5]. At present, studies are mainly focused on conventional starch resources such as cereal and legume seeds, tuber crops, and some root tubers [1,2,6,7]. With the increasing demand for starch, some nonconventional starch resources have been investigated in recent years [3,4,5,8,9].

Banana is an evergreen herbaceous plant that belongs to the genus *Musa* of the family Musaceae. There are over 130 countries and regions producing bananas in the world, mainly in Central and South America and Asia [10]. In 2016, China planted 430,046 hm^2^ and produced 13.324 million tons of bananas [11]. Green banana flesh and peel contain approximate 60% and 30% starch content (from dry mass), respectively [12,13]. The starch is gradually decomposed into soluble sugar under the action of enzymes with the fruit ripeness [14,15,16]. At present, mature banana fruit is mainly used as fresh food for people’s daily life and raw material for processing sugar-based products, and green banana fruit is processed as a starch resource [15,17,18,19].

Banana fruit consists of flesh and peel. To date, research on green banana fruit has mainly focused on the flesh, and contains the investigation of flesh flour and starch. The studies on flesh flour include the effects of different treatments including chemical, physical, and organic acid treatment on the composition, color, water-holding capacity, oil-holding capacity, thermodynamic properties, and static rheological properties of flour, the content and physicochemical properties of resistant starch in flour, and the production process of flour [18,19,20,21,22,23,24]. The studies on flesh starch include its morphology, resistant starch, thermodynamic characteristics, and enzymatic hydrolysis characteristics [19,25,26,27,28]. Studies on green banana peel are mainly focused on the contents of pectin, dietary fiber, carotenoids, phenols, and other active ingredients and their industrial applications [29,30,31,32,33,34]. The total soluble solids, water-holding capacity, oil-holding capacity, and color value of the flour from green banana flesh and peel have been also investigated and compared in previous studies [35]. However, the starch contained in green banana peel is hardly studied and wasted. Therefore, in order to make full use of green banana peel and widen its application, it is necessary to investigate the physicochemical properties of starch from green banana peel.

In this study, starches were isolated from the flesh and peel of green banana fruit. Their morphology, structure, and some functional properties were investigated and compared. Our objectives were to disclose the physicochemical properties of starches from green banana peel and provide useful information for the utilization of green banana peel.

## 2. Results and Discussion

### 2.1. Starch and Soluble Sugar Contents in Dry Flesh and Peel

The contents of soluble sugar and starch in the dry flesh and peel of green banana fruit were determined. The flesh had a slightly lower soluble sugar content (3.4%) than the peel (4.1%); however, the starch content was markedly higher in flesh (69.5%) than in peel (22.6%). The present result agreed with the previous research that the dry flesh of green banana contains approximate 75% starch [12]. The starch content of banana peel on a dry basis was also similar to the previous report ranging from 16% to 48.5% (average 29%) [13]. With the banana fruit gradually maturing, the starch content of flesh drops from approximate 75% to 1% [12], while the content of soluble sugar increases from approximate 1% to 20% [36]. For two banana varieties, Big Ebanga and Grande Naine, the starch content of peel decreases from 39.3% to 0.1% and from 11.1% to 3.3%, respectively, and the soluble sugar content increases from 4.2% to 38.3% and from 2.2% to 32.4%, respectively, as the fruit matures [37]. Compared with mature fruit, the flesh and peel of green banana fruit are an important starch resource.

### 2.2. Morphology and Granule Size of Starch

**Figure 1:**
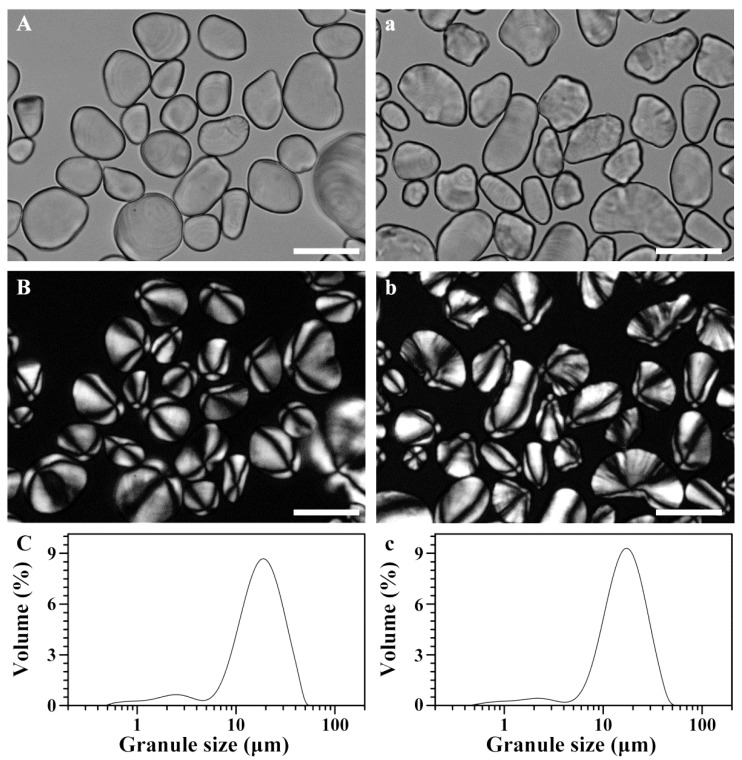
Morphology of starch granules under normal light (**A**,**a**) and polarized light (**B**,**b**), and their size distribution (**C**,**c**). (**A**–**C**), flesh starch; (**a**–**c**), peel starch. Scale bar = 20 μm.

Starch granules isolated from the flesh and peel were observed with a polarized microscope under normal and polarized light (Figure 1). The flesh starch had an oval shape or a globular shape with an eccentric hilum. A similar morphology of granule has been reported in banana flesh [12,27]. The peel starch had some large oval granules and small slender or irregular granules with eccentric hila. Starch granules have stable semi-crystalline structure, and show the Maltese cross under polarized light. The position of hilum and the morphology of the granule are determined by the plant origin [1,2,3,4,5,8,9].

The granule size distribution of starch is shown in Figure 1. Both flesh and peel starches had similar bimodal distribution pattern: a small peak from 0.5 µm to 5 µm, and a large peak from 5 µm to 50 µm. The granule size of peel starch was slightly smaller than that of flesh starch (Table 1). The present granule size of flesh starch was similar to the previous report [38]. The granule size is related to plant origin, amyloplast development, and plant physiology [1,2,3,4,5,8,9], and has important effects on the functional properties of starch [39].

### 2.3. Iodine Absorption Spectrum and Apparent Amylose Content of Starch

**Figure 2:**
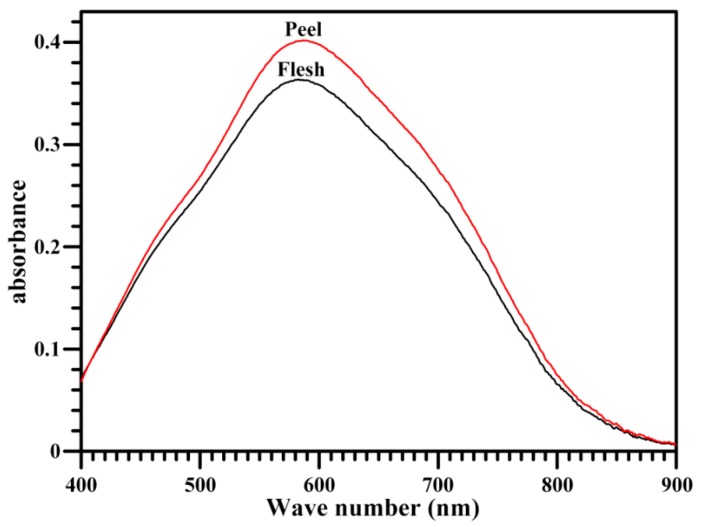
Spectra of iodine absorbance of starches.

The iodine absorption spectrum of starch reflects the content and structural characteristic of starch components—amylose and amylopectin—and its maximum absorption wavelength (λmax) is determined by the chain length of amylose and amylopectin [40]. The iodine absorption spectra were different between flesh and peel starches (Figure 2), and the peel starch had a higher λmax than the flesh starch (Table 2), indicating the difference in starch components between the flesh and peel. The flesh starch had significantly lower apparent amylose content (AAC) (21.3%) than the peel starch (25.7%) (Table 2). The amylose contents ranging from 16% to 40.7% are reported in the flesh starch of banana [12,17,38]. The different amylose contents might result from the different amylose measuring methods, banana varieties, and fruit ripeness.

### 2.4. Crystalline Structure of Starch

**Figure 3:**
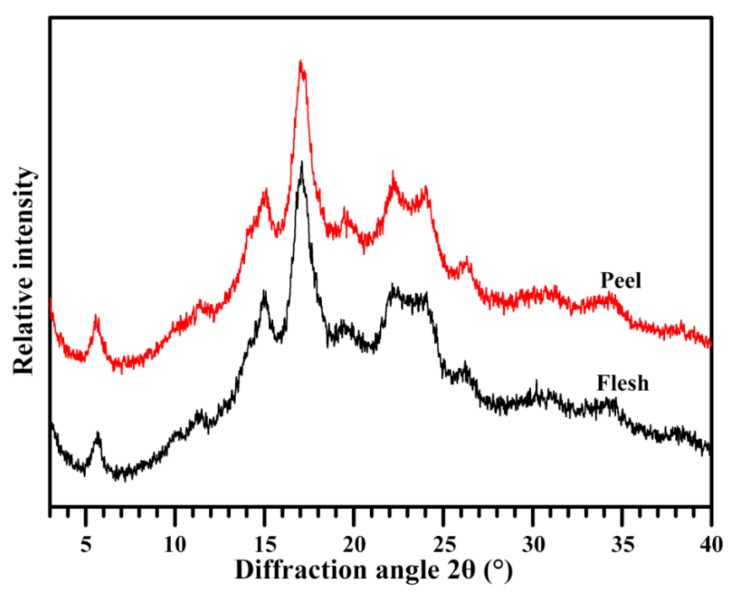
XRD patterns of starches.

The crystalline structure of starch can be detected by X-ray diffraction (XRD). Starches from different plant sources can be divided into A-, B-, and C-type sources according to their XRD patterns [41]. The starches of flesh and peel showed diffraction peaks at about 5.6°, 15°, 17°, 22°, and 24° 2θ, indicating they were B-type starches (Figure 3). The flesh starch had a relative crystallinity of 28.0%, which was slightly higher than that of peel starch (26.1%) (Table 2). Usually, the relative crystallinity is negatively correlated with amylose content [42]. Compared with flesh starch, the low relative crystallinity might result from its high apparent amylose content. In previous studies, the A-, B-, and C-type starches have all been reported in the flesh of banana; their relative crystallinities range from 28% to 30%. The difference in crystalline structure might be caused by the different varieties and growing environments [12,26]. In addition, different measuring methods also influence the relative crystallinity [43].

### 2.5. Short-Range Ordered Structure of Starch

**Figure 4:**
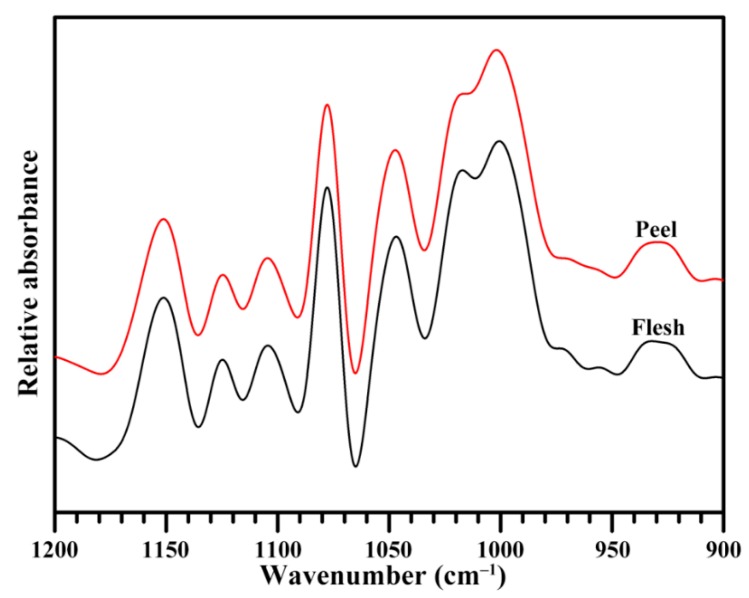
Attenuated total reflectance (ATR)-Fourier transform infrared spectrometer (FTIR) spectra of starches.

Fourier transform infrared spectrometer (FTIR) is often applied for the determination of the short-range ordered structure of starch. The attenuated total reflectance (ATR)-FTIR spectrum can reflect the ordered structure in the external region of the granules [44]. For the ATR-FTIR spectrum, the ratios of 1045/1022 cm^−1^ and 1022/995 cm^−1^ are widely used to quantify the ordered degree and the proportion of amorphous to ordered carbohydrate structure in starch [44]. The starches from banana flesh and peel showed a similar ATR-FTIR spectrum (Figure 4). The ratio of 1045/1022 cm^−1^ was similar between the flesh starch (0.75) and peel starch (0.78), and that of 1022/995 cm^−1^ had also no significant difference between the flesh starch (0.90) and peel starch (0.88), indicating that the flesh and peel starches had a similar short-range ordered structure in the external region of the granule. The ratio of 1045/1022 cm^−1^ of flesh starch in the present study was significantly higher than that of previous study, ranging from 0.55 to 0.60 in two banana varieties [17]. In the report of Jiang et al. [17], the transmission mode of FTIR, which reflects the ordered structure of the whole starch, is used, and the flesh starch has C-type crystallinity. The different analysis mode and starch crystalline structure might result in the different ordered degrees.

### 2.6. Lamellar Structure of Starch

**Figure 5:**
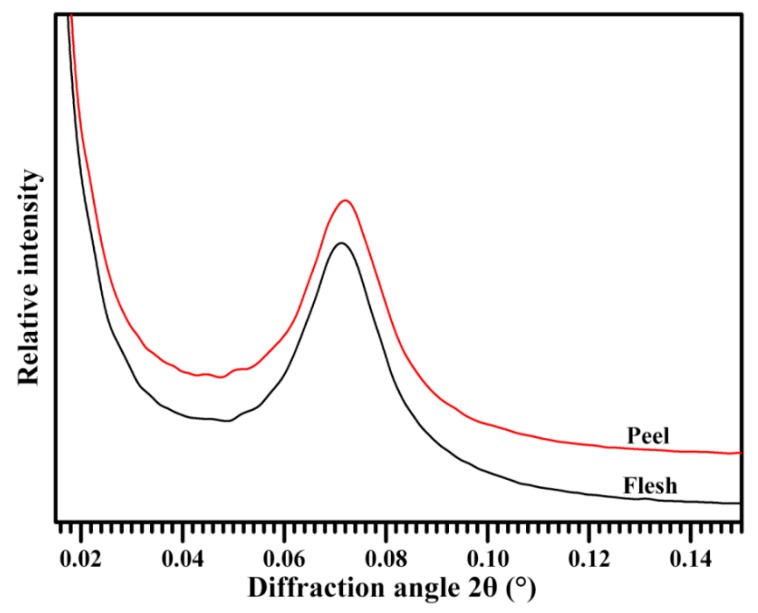
Small-angle X-ray scattering (SAXS) patterns of starches.

Starch is made up of semi-crystalline granules and consists of alternating amorphous and semi-crystalline growth rings. The latter is formed by a lamellar structure that consists of alternating crystalline and amorphous regions. The lamellar structure can be measured by the spectrum of small-angle X-ray scattering (SAXS) [45]. Figure 5 shows the SAXS spectra of flesh and peel starches. In the SAXS spectrum, the scattering peak at approximate 0.07 Å^−1^ is caused by the periodic arrangement of crystalline and amorphous lamellae, corresponding to the lamellar thickness [45]. The peak intensity reflects the difference in electron density between the crystalline and amorphous regions of lamellae, and is negatively related to amylose content [46]. The flesh and peel starch had scattering peak positions at 0.071 and 0.072 Å^−1^, respectively, corresponding to lamellar thicknesses of 8.79 nm and 8.71 nm. The peak intensity was similar between flesh (202.2) and peel starch (199.4). The present results indicated that the starches from flesh and peel had similar lamellar structures.

### 2.7. Thermal Properties of Starch

**Figure 6:**
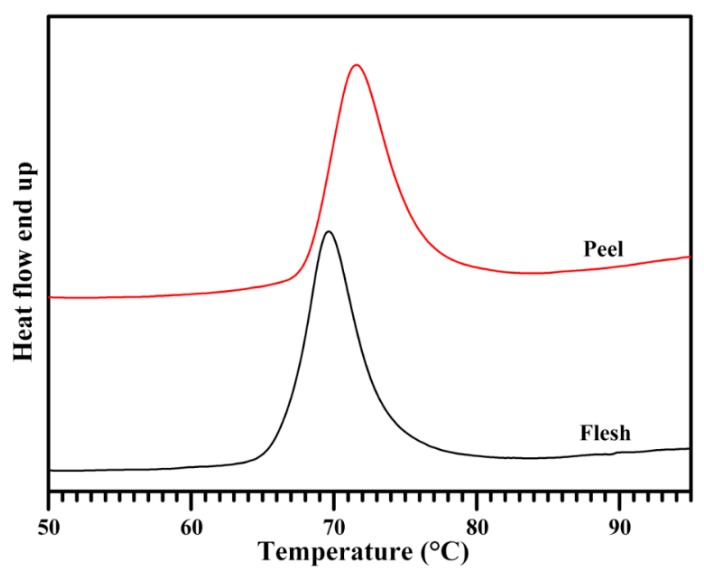
Differential scanning calorimeter (DSC) thermograms of starches.

The thermal properties of starch were measured with a differential scanning calorimeter (DSC). Figure 6 shows the DSC thermograms of starches, and the thermal parameters are presented in Table 3. The gelatinization onset, peak, and conclusion temperatures were significantly higher in peel starch than in flesh starch, while their gelatinization temperature range and gelatinization enthalpy were similar. Utrilla-Coello et al. [38] investigated the thermal properties of flesh starches from different banana varieties, and found that the gelatinization onset, peak, and conclusion temperatures ranged from 60.9 °C to 74.6 °C, from 70.2 °C to 78.7 °C, and from 83.2 °C to 92.4 °C, respectively, and the gelatinization enthalpy ranged from 10.4 to 15.1 J·g^−1^. Starch with high amylose content requires a higher temperature to destroy its internal structure, leading to the gelatinization temperature being positively related to amylose content [38,40,42]. The high gelatinization temperature of peel starch might be relative to its high amylose content.

### 2.8. Swelling Power and Water Solubility of Starch

**Figure 7:**
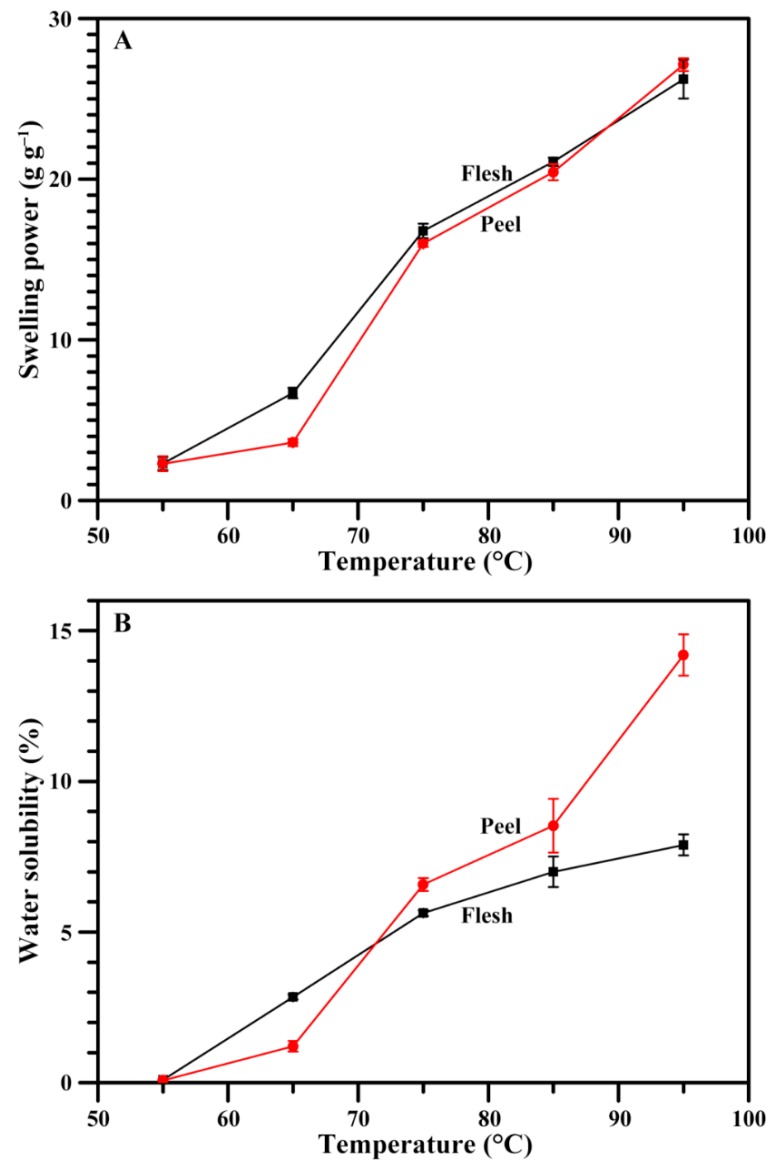
Swelling powers (**A**) and water solubilities (**B**) of starches.

Figure 7 shows the swelling powers and water solubilities of starches at different temperatures. The swelling power is the binding ability of starch and water, and the water solubility reflects the dissolution degree of starch components during granule swelling [7]. The swelling power and water solubility of both flesh and peel starches increased with increasing heat temperature. The swelling power had no difference between flesh and peel starches, but the water solubility of peel starch was significantly higher than that of flesh starch at 95 °C. The structure of amylopectin determines the swelling power of starch, and the amylose content is positively relative to water solubility [38]. The similar swelling powers between flesh and peel starches indicated that they might have similar amylopectin structure, and the high water solubility of peel starch might result from its high amylose content.

### 2.9. Hydrolysis of Starch

**Figure 8:**
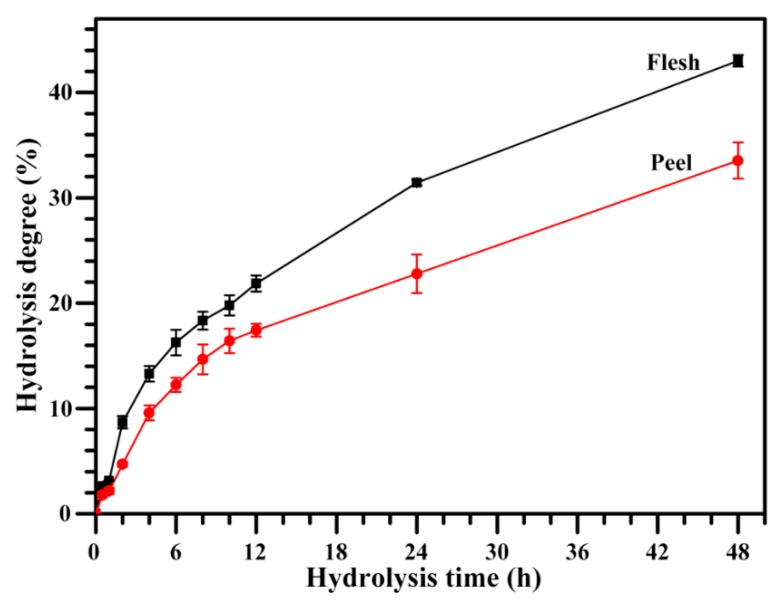
Hydrolysis curves of starches by porcine pancreatic α-amylase (PPA).

Figure 8 shows the dynamic hydrolysis of starch by porcine pancreatic α-amylase (PPA). The rate of hydrolysis was relatively fast at first, and then gradually became slow, showing a diphasic hydrolysis. Similar diphasic hydrolysis dynamics have been reported in previous studies [3,4,5]. Peel starch had a significantly lower hydrolysis degree than flesh starch during hydrolysis, indicating that peel starch had higher resistance to PPA hydrolysis than flesh starch. The enzymatic hydrolysis of starch by PPA is influenced by the starch morphology, granule size, integrity, porosity, crystalline structure, amylose content, and structural heterogeneity [9,39]. The hydrolysis degree of starch is negatively related to granule size and amylose content [39]. In this study, although flesh starch had a slightly higher granule size than peel starch, the significantly higher amylose in peel starch than in flesh starch might result in the slow hydrolysis of peel starch. Normally, the B-type starch is more resistant to enzyme hydrolysis than the A-type starch [47]. However, different B-type starches have also different hydrolysis rates. For example, at the same reaction condition of PPA hydrolysis, the hydrolysis degree of B-type starch for 8 h is approximate 17% for potato [47], 3% for *Curcuma longa*, 13% for *Canna edulis*, 15% for *Canna indica*, 25% for *Lilium lancifolium* [5], 15% for green banana peel, and 18% for green banana flesh (Figure 8). Therefore, starches from different botanical sources had different hydrolysis properties due to their different morphological and structural properties, and it was very important to find new starch resources to meet the demand of starch industries.

### 2.10. Digestion Properties of Starch

The flesh and peel starches were in vitro digested by both PPA and *Aspergillus niger* amyloglucosidase (AAG). Generally, starch can be divided into rapidly digestible starch (RDS), slowly digestible starch (SDS), and resistant starch (RS), according to its digestion rate [48]. The digestion properties of native and gelatinized starches are presented in Table 4. For native starch, peel starch had slightly lower SDS and higher RS contents than flesh starch. For gelatinized starch, the RDS, SDS, and RS contents were similar between flesh and peel starches. Agama-Acevedo et al. [26] measured the RDS, SDS, and RS contents of native starches from the flesh of four banana varieties with different genotype backgrounds; their values ranged from 1.3% to 16.6%, from 4.4% to 18.1%, and from 65.3% to 91.9%, respectively, indicating that the digestion properties of starches are significantly influenced by the varieties. The present results agreed with the report of Agama-Acevedo et al. [26]. The digestion of native starch is related to starch morphology, granule size, crystalline structure, and starch components [17]. The slight difference in the digestion properties of the native starches from the flesh and peel might result from the differences in their granule sizes and amylose contents.

## 3. Materials and Methods

### 3.1. Plant Materials

The fruits of green banana were harvested from the farmland of Lingshui, Hainan Province, China in March 2017. The flesh and peel were separated manually and used as plant materials.

### 3.2. Determination of Starch and Soluble Sugar Contents in Dry Flesh and Peel

The fresh flesh and peel were immediately dried at 110 °C for 3 h and 80 °C for 2 d. The dried flesh and peel were extensively ground into flour in a mortar and pestle, and then passed through a 100-mesh sieve. The contents of soluble sugar and starch in flour were determined according to the experimental method described by Gao et al. [8], with some modifications. Briefly, 100 mg of flour and 5 mL of 80% ethanol were added to a 15-mL glass tube, and then incubated in a water bath at 80 °C for 30 min. The sample was centrifuged at 5000× *g* for 5 min, and the supernatant was transferred to a 100-mL volumetric flask. The precipitate was treated twice more with 80% ethanol, and the supernatant was transferred and made up to 100 mL for measuring the soluble sugar content. The residual precipitate was suspended with 3 mL of deionized water, and incubated in a water bath at 100 °C for 20 min. After cooling to room temperature, 3 mL of 9.2 M HClO_4_ was added to the sample for 15 min. The sample was centrifuged at 5000× *g* for 5 min, and the supernatant was transferred to a 100-mL volumetric flask. The precipitate was treated twice more with 6 mL of 4.6 M HClO_4_, and the supernatant was transferred and made up to 100 mL for measuring the starch content. The soluble carbohydrates in the above supernatants were determined using an anthrone–H_2_SO_4_ method, and converted to the contents of soluble sugar and starch in flour.

### 3.3. Starch Isolation

The starches were isolated from fresh flesh and peel according to the methods described by Waliszewski et al. [49] and Fan et al. [4], with some modifications. Briefly, fresh and peel were cut into small pieces and homogenized in sodium bisulfite solution (0.025%, *w*/*w*) using a home blender (JYL-C93T, Joyoung, Suzhou, Jiangsu, China). The resultant slurry was squeezed through four layers of cheesecloth and sieved through a 100-mesh and 200-mesh screen. The starch suspension was left overnight at 4 °C. The precipitated starch was washed three times with NaOH aqueous solution (0.2%, *w*/*w*), three times with deionized water, and twice with anhydrous ethanol by centrifugation. During water washing, the dirty gel-like layer on top of the white starch precipitate was carefully scraped off and discarded. Finally, the starch was dried at 40 °C, ground into powder, and passed through a 100-mesh sieve.

### 3.4. Morphology Observation and Granule Size Analysis

The 10-mg starch was suspended in 1 mL of 50% glycerol, and a 10-µL suspension was placed on the microscope slide and covered with a coverslip. The morphology of starch granules was observed and photographed using a polarized microscope (BX53, Olympus, Tokyo, Japan) under normal and polarized light. The starch granule size was analyzed using a laser diffraction particle analyzer (Mastersizer 2000, Malvern, Worcestershire, UK) according to the method described by Cai et al. [50].

### 3.5. Determination of Iodine Absorption Spectrum and AAC

The 10 mg of starch and 5 mL of dimethyl sulfoxide containing 10% 6 M urea were added to a 10-mL glass tube, and then incubated in a water bath at 95 °C for 1 h with interval vortexing. After cooling to room temperature, 1 mL of sample solution and 1 mL of 0.2% iodine solution were added and made up to 50 mL in a volumetric flask with deionized water. The sample was held in dark for 20 min. The iodine absorbance spectrum was scanned using a spectrophotometer (Ultrospec 6300 pro, Amershan Bioscience, Cambridge, Sweden). The AAC was determined from the absorbance at 620 nm.

### 3.6. Crystalline Structure Analysis

The crystalline structure of starch was investigated using an X-ray powder diffractometer (D8, Bruker, Karlsruhe, Germany), following our previously described method [51]. Briefly, starch powder was wetted in a desiccator where a saturated NaCl solution maintained a humidity atmosphere for one week at room temperature. The starch powder was flattened on the sample stage, and scanned from 3° to 40° 2θ with a step size of 0.02°, a count time of 0.8 s, and an X-ray beam at 200 mA and 40 kV. The relative crystallinity was the area percentage of crystalline diffraction peak area to total diffraction area between 4° and 30° 2θ.

### 3.7. Short-Range Ordered Structure Analysis

The short-range ordered structure of starch was measured using an FTIR spectrometer (7000, Varian, Santa Clara, CA, USA) with a deuterated triglycine sulphate (DTGS) detector equipped with an ATR cell following our previously described method [51]. Briefly, the starch–water slurry was added to the sample cell, and scanned from 4000 cm^−1^ to 800 cm^−1^ with 4 cm^−1^ resolution and 64 scans. The spectrum was corrected by a baseline between 1200 cm^−1^ and 800 cm^−1^; then, the deconvolution was performed using Resolutions Pro with a Lorentzian line shape, a half-width of 19 cm^−1^, and a resolution enhancement factor of 1.9.

### 3.8. Lamellar Structure Analysis

The lamellar structure of starch was measured following our previously described method [45]. Briefly, 200 mg of starch powder was immersed in 1 mL of deionized water overnight at 4 °C. The sample was centrifuged at 3000× *g* for 5 min, and the starch precipitate was sealed in a sample cell to prevent dehydration. The sample was measured using a SAXS instrument (NanoStar, Bruker, Karlsruhe, Germany) equipped with a Vantec 2000 detector and pinhole collimation for point focus geometry. The X-ray source was a copper rotating anode (0.1 mm filament) at 50 kV and 30 W, and the Cu Kα radiation wavelength was 1.5418 Å. During analysis, the sample was under vacuum to avoid the air scattering. The SAXS dataset was analyzed using DIFFRAC^plus^ NanoFit software (NanoStar, Bruker, Karlsruhe, Germany). The spectrum parameters were measured using the simple graphical method [45].

### 3.9. Determination of Swelling Power and Water Solubility

The swelling power and water solubility of starch were measured according to the method described by Lin et al. [52], with some modifications. Briefly, 30 mg of starch and 1.5 mL of deionized water were added to a 2-mL centrifuge tube. The sample was incubated in a ThermoMixer C (Eppendorf, Hamburg, Germany) at different temperatures with continuous shaking (1000 rpm) for 30 min. After incubation, the sample was cooled to room temperature and centrifuged at 8000× *g* for 10 min. The weight of the original starch was recorded as W1, the starch weight in supernatant was determined by the anthrone–H_2_SO_4_ method and recorded as W2, and the mass of precipitate obtained after centrifugation was recorded as W3. The swelling power was obtained by W3/(W1 − W2) × 100%, and the water solubility was obtained by W2/W1 × 100%.

### 3.10. Determination of Thermal Properties

The 5 mg of starch was accurately weighed in an aluminum pan (Netzsch, Selb, Germany), and then added to 15 µL of deionized water. The sample was mixed, sealed, and held at 4 °C overnight. After balancing at room temperature for 1 h, the sample was heated from 25 °C to 130 °C at 10 °C/min using a differential scanning calorimeter (200-F3, Netzsch, Selb, Germany)

### 3.11. Determination of Hydrolysis Degree

The starch was hydrolyzed by PPA (A3176, Sigma Aldrich, St. Louis, MO, USA) according to the method described by Gao et al. [8], with some modifications. Briefly, the 10-mg starch was suspended in 2-mL enzyme solution (0.1 M of sodium phosphate buffer, pH 6.9, 25 mM of NaCl, 0.01% NaN_3_, 5 mM of CaCl_2_, 50 U PPA). The sample was incubated in a ThermoMixer C (Eppendorf, Hamburg, Germany) at 37 °C and 1000 rpm for 0.5 h, 1 h, 2 h, 4 h, 6 h, 8 h, 10 h, 12 h, 24 h, or 48 h. After reaction, the sample was centrifuged at 8000× *g* for 5 min, and the soluble sugar in the supernatant was measured using an anthrone–H_2_SO_4_ method and converted to the hydrolyzed starch.

### 3.12. Determination of Digestion Properties

The digestion properties of native and gelatinized starches were determined according to the previous method described by Lin et al. [40], with some modifications. Briefly, 10 mg of starch and 1 mL of deionized water were added to a 2-mL centrifuge tube, and incubated in a ThermoMixer C (Eppendorf, Hamburg, Germany) at 98 °C and 1000 rpm for 12 min to prepare the gelatinized starch. Then, to native or gelatinized starch slurry was added 1 mL of enzyme solution (40 mM of sodium phosphate buffer, pH 6.0, 13.4 mM of NaCl, 0.02% NaN_3_, 5 mM of CaCl_2_, 4 U PPA (A3176, Sigma Aldrich, St. Louis, MO, USA), 4 U AAG (E-AMGDF, Megazyme, Bray, Ireland)). The sample was incubated in a ThermoMixer C (Eppendorf, Hamburg, Germany) at 37 °C and 1000 rpm for 20 min or 2 h. After reaction, the sample was immediately transferred to a 10-mL glass tube with 2 mL of 50% ethanol and 240 µL of 0.1 M HCl, and centrifuged at 8000× *g* for 5 min. The glucose content in the supernatant was measured with a glucose assay kit (K-GLUC, Megazyme, Bray, Ireland) and converted the degraded starch. The RDS, SDS, and RS were obtained according to the degraded starch within 20 min, between 20 min and 2 h, and after 2 h, respectively.

### 3.13. Statistical Analysis

All of the data in tables are given as means ± SD and analyzed with Student’s *t*-test.

## 4. Conclusions

The dry flesh and peel of green banana contained 69.5% and 22.6% starch content, respectively, indicating that they are an important starch resource. Flesh and peel starches had oval and irregular granules with eccentric hila. The granule size of peel starch was slightly lower than that of flesh starch. The λmax and AAC were higher in peel starch than in flesh starch. Both flesh and peel starches had B-type crystalline structures and similar relative crystallinity, short-range ordered structures in the external region of the granules, and lamellar structures. The swelling power was similar between the flesh and peel starches, but the water solubility of peel starch was higher at 95 °C than that of flesh starch. The gelatinization temperature was higher in peel starch than in flesh starch, but their gelatinization enthalpy was similar. The digestion properties of native and gelatinized starches were similar between flesh and peel starches, except that the peel native starch had slightly lower SDS and higher RS contents than flesh native starch. This study could provide important information for the applications of the peel starch of green banana fruit.

## Figures and Tables

**Table 1:** Granule sizes of starches.

Tissues	d(0.1) (μm)	d(0.5) (μm)	d(0.9) (μm)	D[4,3] (μm)
Flesh	6.949 ± 0.001	16.439 ± 0.016	30.066 ± 0.034	17.496 ± 0.017
Peel	7.450 ± 0.002 ***	15.355 ± 0.001 ***	27.249 ± 0.005 ***	16.353 ± 0.001 ***

The d(0.1), d(0.5), and d(0.9) are the diameter of granules for which 10%, 50%, and 90% of particles are smaller by volume, respectively. The D[4,3] is the mean diameter by volume. Data are mean ± standard deviations for triplicate. * The data show significant difference between flesh and peel starches (*** *p* < 0.001).

**Table 2:** Maximum absorption wavelengths (λmax), apparent amylose contents (AAC), and relative crystallinities (RC) of starches.

Tissues	λmax (nm)	AAC (%)	RC (%)
Flesh	584.5 ± 0.9	21.3 ± 0.3	28.0 ± 0.8
Peel	586.5 ± 0.5 *	25.7 ± 0.3 ***	26.1 ± 0.5

Data are means ± standard deviations for triplicate. * The data show significant difference between flesh and peel starches (* *p* < 0.05 and *** *p* < 0.001).

**Table 3:** Thermal parameters of starches.

Tissues	To (°C)	Tp (°C)	Tc (°C)	ΔT (°C)	ΔH (J·g^−1^)
Flesh	66.4 ± 0.1	69.8 ± 0.1	74.0 ± 0.6	7.6 ± 0.5	15.8 ± 0.6
Peel	68.0 ± 0.1 ***	71.6 ± 0.1 ***	76.4 ± 0.3 **	8.4 ± 0.2	16.1 ± 0.8

To, Tp, and Tc: gelatinization onset, peak, and conclusion temperature, respectively; ΔT and ΔH: gelatinization temperature range (Tc–To) and enthalpy, respectively. Data are means ± standard deviations for triplicate. * The data show significant difference between flesh and peel starches (** *p* < 0.01 and *** *p* < 0.001).

**Table 4:** Digestion properties of starches.

Tissues	Native Starch			Gelatinized Starch		
	RDS (%)	SDS (%)	RS (%)	RDS (%)	SDS (%)	RS (%)
Flesh	1.7 ± 0.2	4.3 ± 0.3	94.1 ± 0.2	73.0 ± 1.1	5.1 ± 0.2	21.9 ± 1.2
Peel	1.4 ± 0.3	3.4 ± 0.2 *	95.2 ± 0.3 *	72.3 ± 0.7	4.5 ± 1.0	23.2 ± 0.5

RDS: rapidly digestible starch; SDS: slowly digestible starch; RS: resistant starch. Data are mean ± standard deviations for triplicate. * The data show significant difference between flesh and peel starches (* *p* < 0.05).
